# Safety and Efficacy of Injecting Mesenchymal Stem Cells Into a Human Knee Joint To Treat Osteoarthritis: A Systematic Review

**DOI:** 10.7759/cureus.24823

**Published:** 2022-05-08

**Authors:** Shoukrie I Shoukrie, Sathish Venugopal, Ravneet K Dhanoa, Ramaneshwar Selvaraj, Tharun Y Selvamani, Anam Zahra, Jyothirmai Malla, Ranim K Hamouda, Pousette F Hamid

**Affiliations:** 1 Orthopaedics and Traumatology, California Institute of Behavioral Neurosciences & Psychology, Fairfield, USA; 2 Neurosurgery, California Institute of Behavioral Neurosciences & Psychology, Fairfield, USA; 3 Internal Medicine, California Institute of Behavioral Neurosciences & Psychology, Fairfield, USA; 4 Family Medicine, California Institute of Behavioral Neurosciences & Psychology, Fairfield, USA; 5 Surgery, California Institute of Behavioral Neurosciences & Psychology, Fairfield, USA; 6 Neurology, California Institute of Behavioral Neurosciences & Psychology, Fairfield, USA

**Keywords:** injection, intra-articular, mesenchymal stem cells, osteoarthritis, knee

## Abstract

Intraarticular stem cell therapy has become increasingly used to treat knee osteoarthritis (KOA) with minimal high-quality evidence to support its use. This study aims to see how well intra-articular injections of mesenchymal stem cells (MSCs) worked and how safe they were for individuals with KOA. A total of 10 studies were extracted using PubMed, Cochrane Library, and PMC from 2017 to 2021 in the English language. An assessment of the risk of bias was applied via the Cochrane Collaborative Bias Risk Tool and Newcastle-Ottawa Quality. Changes in pain and functional outcomes in patients with KOA were measured by a Knee injury and Osteoarthritis Outcome Score (KOOS) scores, Visual Analogue Scale (VAS), Western Ontario and McMaster Universities Osteoarthritis Index (WOMAC) scores at baseline, and follow-up evaluation criteria. The magnetic resonance imaging (MRI) was evaluated using the whole-organ magnetic resonance imaging score (WORMS) and cartilage volume changes. A total of six randomized controlled trials (RCTs), three prospective retrospective clinical trials, and one retrospective clinical trial included 723 patients. They were diagnosed with unilateral or bilateral KOA with Kellgren-Lawrence (KL) grade 1-4 KOA and followed up for six, 12, and 24 months. The experimental groups received multipotent MSCs, mesenchymal progenitor cells (MPCs), adipose tissue progenitor stem cells (AD-MPCs), adipose tissue mesenchymal stem cells (AD-MSCs), bone marrow mesenchymal stem cells (BM-MSCs), bone marrow aspiration (BMA), bone marrow aspiration concentration (BMAC), or micro fragmented adipose tissue (MFAT) while the controlled groups received normal saline (NS), hyaluronic acid (HA), placebo, or went through conservative management.

In conclusion, significant improvements were noticed in the MSCs groups via different outcome measuring tools like KOOS, VAS, WOMAC, and MRI. Furthermore, no significant adverse events (AEs) have been observed. Therefore, intra-articular injections of MSCs are effective and safe in relieving pain and improving motor function in individuals with KOA in the short term, contrary to earlier research findings.

## Introduction and background

Osteoarthritis (OA) is the most common type of arthritis [1], and it is characterized by a progressive loss of articular cartilage, subchondral bone edema, sclerosis, synovitis, and marginal osteophyte formation. Pain, stiffness, and a restriction in joint movement are the most common symptoms whose severity varies. However, the condition gradually worsens over time and often results in significant functional impairment and reduced quality of life [2,3]. It was anticipated to become the fourth leading cause of disability by 2020 [1,4,5], posing a significant socioeconomic burden impacting developed countries' gross domestic product [1,6]. Knee osteoarthritis (KOA) accounts for 85 percent of the global burden of OA and affects 19% of adults over 45-year-old and 37% of people over 60. KOA produces significant pain and physical impairment, lowering the quality of life and ranking as the eleventh leading cause of global disability. The average annual total expense per KOA patient is over US$15 000, resulting in total healthcare expenditure of nearly US$34 billion. Given population aging and the rise in obesity, KOA healthcare expenses are expected to quadruple by 2040 [7]. It is necessary to develop sufficient medicines capable of slowing the progression of the disease and, as a result, preventing the loss of articular function and joint replacement. To provide more effective therapies, current conservative choices such as exercise and physiotherapy and weight loss with analgesics and naturally occurring substances should be integrated [1,8]. Developing effective conservative methods would be especially important for treating young people with early OA because their more active and physically demanding lifestyle negatively correlates with prosthetic implant survival [1,9]. 

The main treatment in the clinic is non-steroidal anti-inflammatory drugs (NSAIDs), which are recommended for all patients except those having surgical treatment in the American Academy of Orthopaedic Surgeons (AAOS) clinical practice recommendations for KOA treatment [10-12]. However, long-term usage of these treatments will cause major adverse reactions in patients, such as gastrointestinal ulcers, digestive system hemorrhage, and cardiovascular and cerebrovascular side effects, regardless of the toxicity of the drugs themselves [10,13]. Intra-articular injections of HA, platelet-rich plasma (PRP), or corticosteroids (CC) are also clinical possibilities, but their efficacy and the prevalence of side effects are still debated [10,14,15].

MSCs, be a possible treatment option for KOA [16-20]. MSCs, also called MPCs, secrete various cytokines that modulate an anti-inflammatory milieu in the OA joint, giving them immunomodulatory characteristics [18,21]. They may also have a unique ability to induce the growth of new cartilage-like cells in vitro [17,18,22], as improvements in cartilage morphology have been found in some situations [23-26]. These characteristics make them a suitable candidate for use in knee cartilage repair [27-32]. For OA treatment, orthobiologics injections containing MSCs as effector cells have recently been used. Because of their accessibility, bone marrow (BM) and adipose tissue (AD) have traditionally been the most used autologous tissue sources for orthopedic usage. In several studies, the use of autologous orthobiologics treatments in the treatment of OA is safe, with an extensive multicenter prospective analysis revealing no higher risk of neoplasia [33,34].

MSCs treatment looks to be safe based on published clinical study results. There were no significant side effects other than transitory fever in a comprehensive systematic review and meta-analysis of trials involving intravascular delivery of autologous or allogeneic expanded MSCs treatments (totaling over 1000 participants) [35,36]. A systematic evaluation of clinical trials involving intra-articular autologous expanded MSCs therapy that included 844 procedures. They had a mean follow-up of 21 months and found no link between infection, cancer, or death [35,37]. 

As a result, we undertook this study to examine all current high-quality information on the therapeutic efficacy and safety of MSCs in the treatment of KOA qualitatively and quantitatively. This is crucial, and the study's findings will give evidence and recommendations for the promotion and deployment of MSCs therapy in clinical practice. 

## Review

Method

We developed and implemented the study according to the Preferred Reporting Items for Systematic reviews and Meta-Analyses (PRISMA) system [38], the review's preferred reporting items.

Database 

On December 30, 2021, we began our research using online libraries as a database. For our data gathering, we used PubMed, the Cochrane Library, and PMC.

Search Strategy

We included studies related to KOA, MSCs, and intra-articular injection. Our keywords and medical subject heading (MeSH) search strategies included knee, osteoarthritis, mesenchymal stem cells, intra-articular, and injection. The main MeSH terms used were: ("injections, intra articular"[MeSH Terms] OR ("injections"[All Fields] AND "intra articular"[All Fields]) OR "intra-articular injections"[All Fields] OR ("intra"[All Fields] AND "articular"[All Fields] AND "injection"[All Fields]) OR "intra articular injection"[All Fields]) AND ("mesenchymal stem cells"[MeSH Terms] OR ("mesenchymal"[All Fields] AND "stem"[All Fields] AND "cells"[All Fields]) OR "mesenchymal stem cells"[All Fields]) AND ("osteoarthritis, knee"[MeSH Terms] OR ("osteoarthritis"[All Fields] AND "knee"[All Fields]) OR "knee osteoarthritis"[All Fields] OR ("knee"[All Fields] AND "osteoarthritis"[All Fields])) and “Knee Osteoarthritis”, “Mesenchymal Stem Cells”, “Intra-articular Injections”. MeSH terms carried out a further supplementary search with free words. In addition, to prevent eliminating papers that satisfied the inclusion criteria, we searched retrieved studies that were cited. 

Inclusion Criteria

We included RCTs and clinical trial studies conducted between 2017-and 2021, with complete free texts in the English language from all countries. Also, men and women aged 18 years or older with osteoarthritis in their knees and the severity of their osteoarthritis are shown in KL grade. 

Exclusion Criteria

We excluded studies before the last five years, not in English, that included animals, HA, PRP, arthroscopy, ultrasound waves, and combination treatment in the intervention, other than knee joints like shoulder and hip.

Quality Assessment Tools

Two authors, S.S and S.V, independently assessed the study's overall quality and risk of bias by using the Cochrane Collaboration risk-of-bias tool for the RCTs and Newcastle Ottawa Scale (NOS) for the clinical trials. The Cochrane Collaboration risk-of-bias tool included random sequence generation, allocation concealment, blinding of participants and personnel, blinding of outcome assessment, incomplete outcome data, selective reporting, and other biases. Each included RCT was rated as having a low, unclear, or high risk of bias based on these factors. The following are the contents for the NOS, including selection, comparability, and outcome. According to these items, each included clinical trial was scored as good, fair, and poor quality.

Data Extraction

Two writers, S.S and S.V, worked independently to extract data using a standardized manner. Disagreements that arose during the procedure were resolved through debate between the two writers or contact with a third author, just as they were with the inclusion of literature into the study. The following were the contents of the data extraction form: the first author's name, the year of publication, the sample size, basic patient information (age, male-to-female ratio, body mass index (BMI)), osteoarthritis grading KL grade, donor source (autogenous/allogeneic), cell processing, culture, and harvesting, number of cells, immunophenotype, intervention, and control situation, follow-up, and outcome clinical effectiveness and safety were among the outcomes.

Results 

Literature Search

**Figure 1:**
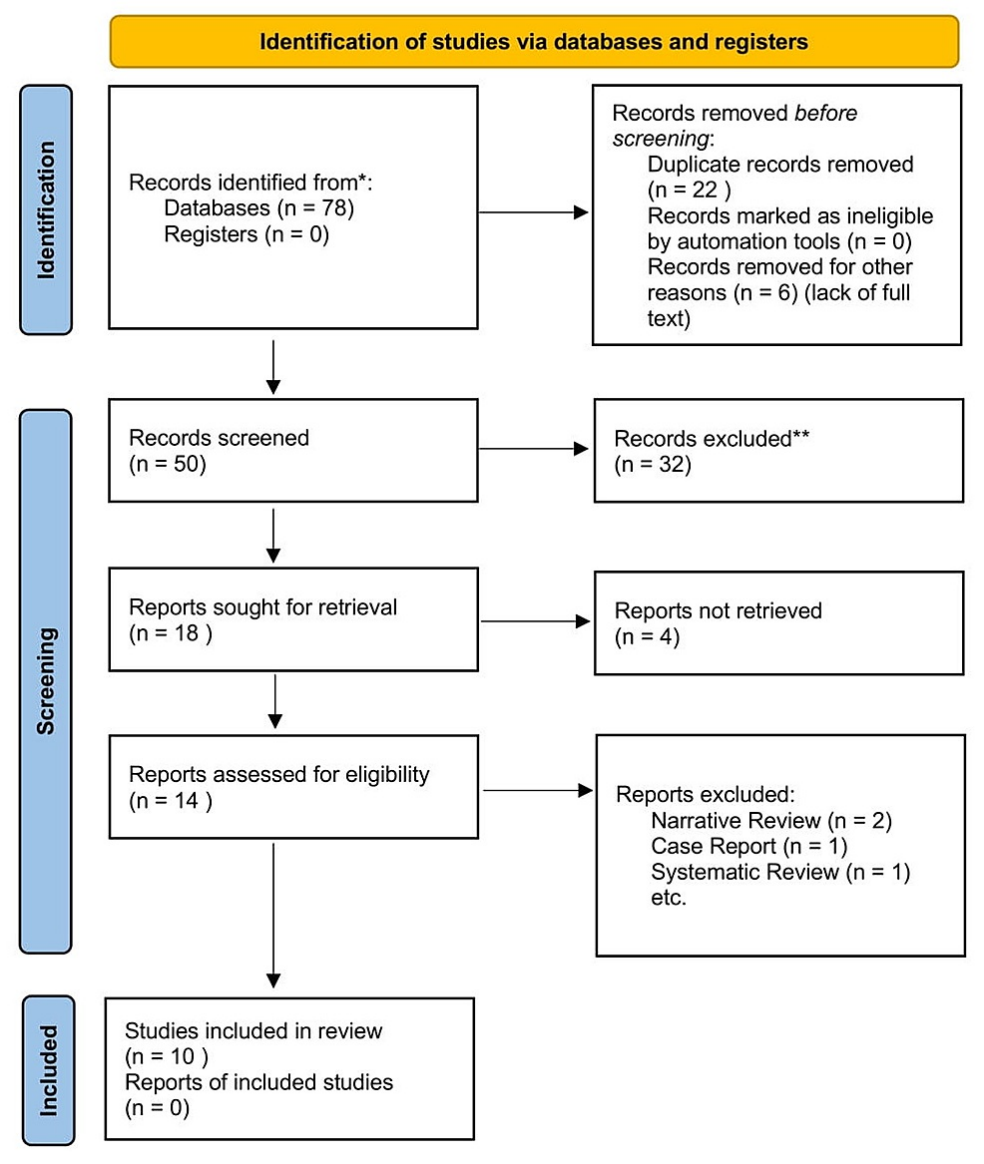
The literature screening process is strictly according to the inclusion/exclusion criteria. RCT and Clinical Trials

Using the literature search, we discovered 78 relevant papers. After eliminating duplicates and screening titles and abstracts, 50 articles were excluded. The remaining 18 articles were subjected to a full-text review, with eight being excluded, as shown in figure 1.

Characteristics of the Included Studies

A total of six RCTs (577 participants) [2,7,17,18,32,35], including one study which had a pilot study, commenced in November and completed in June 2021, where recruitment commenced in January and August 2021 and will be finished by December 2024 [7]. Four clinical trial studies, including three prospective [16,23,32], and one retrospective [33] clinical trial, were included in this systematic review. Publication intervals for all 10 were from 2017 to 2021 [7]. All studies used autologous MSCs except two studies [2,7], which used allogeneic MSCs. Five studies [2,17,18,35,39], used AD-MSCs two studies [23,32], used BM-MSCs, one study [16], used BMA, one study [33], used both concentrations BMAC and MFATand one study [7], used multipotent MSCs. A placebo was utilized as a control group [2,39]. For one study, NS was used as the control group [7]; for one trial, HA was used as the control group [17], In one study's control group, cautious management was adopted [35], and five of the investigations [16,18,23,32,33], were uncontrolled. Furthermore, four trials [2,16,17,35] were monitored for a year, three trials [7,23,32] were monitored for 24 months, and two trials [33,39] were followed for six months after they were completed, and one study [18], had a 48-weeks follow-up period. Table 1 illustrates the features of the 10 articles that were featured.

**Table 1:** Features of the included studies. BMI = body mass index, KL = Kellgren-Lawrence, RCT = randomized control trial, MSCs = mesenchymal stem cells, NS = normal saline, CD = cluster of differentiation, KOOS = knee injury and osteoarthritis outcome score, MOAKS = MRI osteoarthritis knee Score, AD-MPCs = adipose tissue mesenchymal progenitor cells, WOMAC = Western Ontario and McMaster universities osteoarthritis index, VAS = visual analogue scale, WORMS = whole-organ magnetic resonance imaging score, MRI = magnetic resonance imaging, HA = hyaluronic acid, AD- MSCs = adipose tissue mesenchymal stem cells, NPRS = numeric pain rating scale, BMAC = bone marrow aspiration concentration, MFAT = microfragmented adipose tissue

Study (year)	Sample (M:F)	Age	BMI	Kl Grade	Type of Study	Intervention	Control	Donor	Immunophenotype	Dose (cell) (×10^6^)	Outcome Measures	Follow up
Liu (2021 [7]	440 (Not mentioned)	40-90	Not mentioned	2-3	Double-Blinded RCT	multipotent MSCs 220	NS, 220	Allogeneic	CD34	2.5×10	KOOS, MOAKS, others	24 months
Lu (2020)[18]	22 (3:19)	18-70	27.77 (±1.93), 26.69 (±2.63), 24.51 (± 2.49)	2-3	Double-Blinded RCT	AD-MPCs 22	Not Controlled	Autologous	Positive marker (CD90, CD73, CD105) Negative (HLA-DR, CD14, CD45)	1 × 10, 2 × 10, 5 × 10	WOMAC, VAS, WORMS, MRI, others	48 weeks
Lu (2019)[17]	52 (6:46)	18–70	24	1-3	Double-Blinded RCT	AD-MPCs 26	HA, 26	Autologous	Positive: CD90,CD73, CD29, CD49 Negative: CD14,CD34,CD45, HLA-DR	50 × 2	WOMAC, VAS, MRI, others	12 Months
Lee (2019)[39]	24 (6:18)	18-75	25.3 (± 4.9), 25.4 (± 3.0)	2-4	Double-Blinded RCT	AD-MSCs 12	Placebo, 12	Autologous	Positive: CD90,CD73 Negative: CD31, CD34, CD45	100	KOOS, WOMAC, VAS, MRI, others	6 months
Freitag (2019) [35]	30 (16:14)	> 18	25.2 (±3.4), 31.6 (±5.9), 30.4 (±5.6)	2-3	Non-Blinded RCT	AD-MSCs 20	Conservative Management, 10	Autologous	Positive: CD90,CD73, CD105 Negative: CD14,CD19, CD34, CD45	100, 100 × 2	KOOS, NPRS, WOMAC, others	12 months
Kuah (2018) [2]	20 (12:8)	40–65	20-30	1-3	Double-Blinded RCT	AD-MSCs 16	Placebo, 4	Allogeneic	Not mentioned	3.9, 6.7	VAS, WOMAC, MOAKS, MRI, others	12 months
Al-Najar (2017) [32]	13 (6:7)	34–63	Not mentioned	2-3	Prospective Clinical Trial	BM-MSCs 13	Not Controlled	Autologous	Positive: CD90, CD105, CD73, CD44 Negative: CD34, CD45, CD11b, CD19, HLA-DR	30.8, 30.4	KOOS, MRI, others	24 months
Chahal (2019) [23]	12 (7:5)	40-65	Not mentioned	3-4	Prospective Clinical Trial	BM-MSCs 12	Not Controlled	Autologous	Positive: CD90, CD105, CD73 Negative: CD45, CD34, CD19, CD14, HLA-DR	1, 10, 50	KOOS, WOMAC, WORMS, others	24 months
Wells (2021) [16]	10 (4:6)	18–79	Not mentioned	1-2	Prospective Clinical Trial	BMA-MSCs 11	Not Controlled	Autologous	Positive: CD90, CD73, and CD105 Negative: CD19, CD34, CD45, CD11b, and HLA-DR	9.9±1.2 / ml (without × 10^6^)	KOOS, NRSP, others	12 months
Mautner (2019) [33]	76 (36:40)	52-74	Not mentioned	1-4	Retrospective Clinical Trial	BMAC 41, MFAT 35	Not Controlled	Autologous	Not mentioned	BMAC 8 cc, MFAT 30 cc (without × 10^6^)	KOOS, VAS, MRI, others	6 months

Risk of Bias Assessment

**Figure 2:**
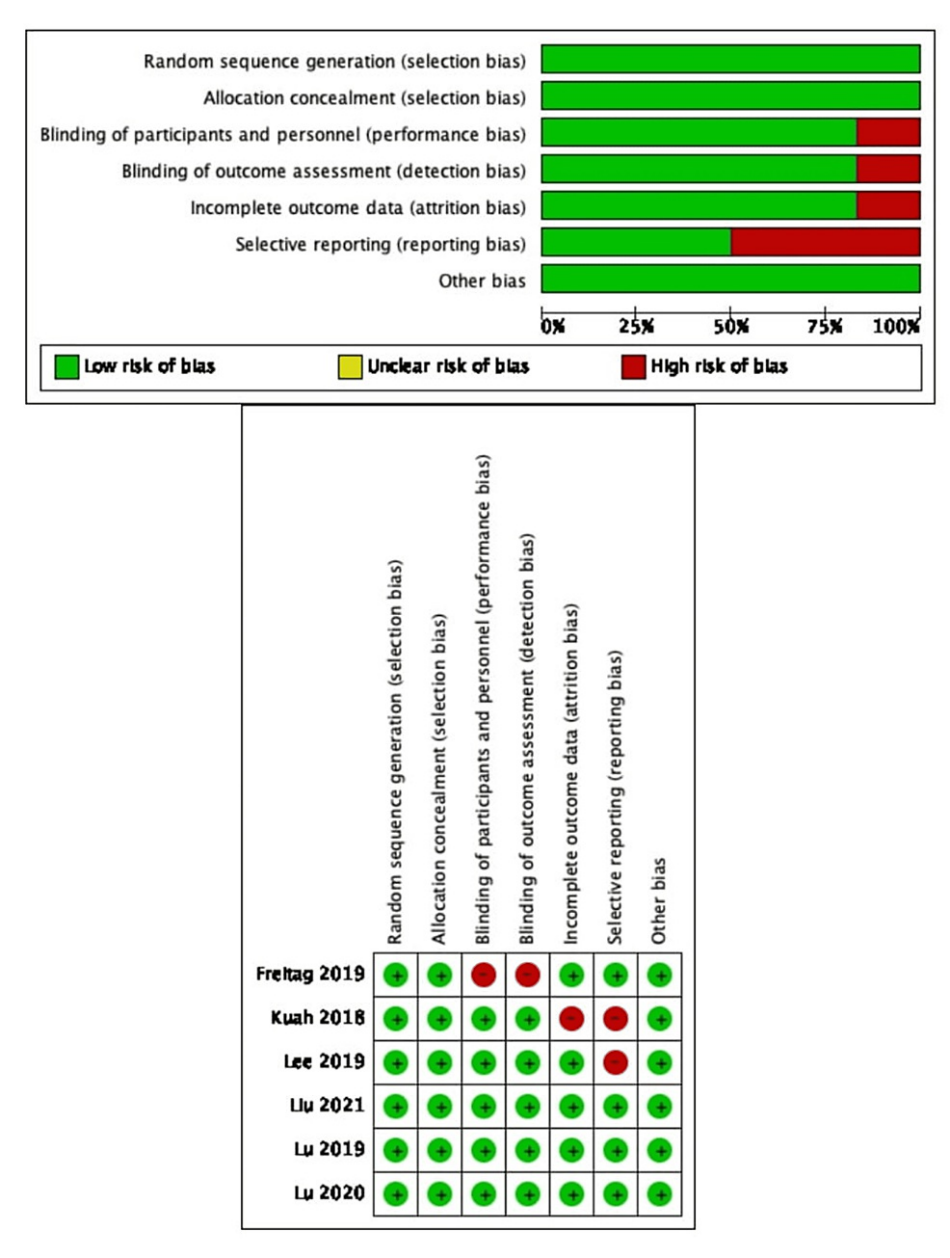
Summary of the risk of bias assessment for the RCT included studies Freitag (2019) [35], Kuah (2018) [2], Lee (2019) [39], Liu (2021) [7], Lu (2019) [17], and Lu (2020) [18]

Figure 2 shows the results of the risk of bias evaluation for six studies [2,7,17,18,35,39], while table 2 shows the results of the NOS for four studies [16,23,32,33]. Lee et al. [39], although relevant images were drawn, we could not retrieve the original data and conduct the combined statistics; hence this study was classified as having a high risk of reporting bias. Freitag et al. and Kuah et al. incomplete data on overall WOMAC scores and subscales (pain, stiffness, and function) were also given, and one or more of these characteristics may have been missing. As a result, attrition bias was found to be considered a risk in these two investigations [2,35]. Freitag et al. performed BM or subcutaneous tissue extraction only in the intervention group. Even though moral restraint precluded the same measures from being used in the control group, this study was classified as having a high risk of detection and performance bias [35].

**Table 2:** Summary of the Newcastle Ottawa Scale for the included clinical trials studies Good quality: 3 or 4 stars in selection domain AND 1 or 2 stars in comparability domain AND 2 or 3 stars in outcome/exposure domain Fair quality: 2 stars in selection domain AND 1 or 2 stars in comparability domain AND 2 or 3 stars in outcome/exposure domain Poor quality: 0 or 1 star in selection domain OR 0 stars in comparability domain OR 0 or 1 stars in outcome/exposure domain

#	Study (Year)	Selection	Comparability	Outcome	Overall Grade
1	Al-Najar et al. (2017) [32]	***	*	**	Good
2	Chahal et al. (2019) [23]	***	*	***	Good
4	Mautner et al. (2019) [33]	***	*	**	Good
3	Wells et al. (2021) [16]	***	*	***	Good

Outcomes

Knee Injury and Osteoarthritis Outcome Score (KOOS): A total of seven studies [7,16,23,32,33,35,39] reported KOOS [40] at baseline and final follow-up in the intervention and the control groups, including 650 patients. Three studies [7,23,32] were followed up for 24 months, two studies [16,35] were followed up for 12 months, and two studies [33,39] were followed up for six months. Normalized KOOS was used to measure positive changes in all five primary areas, and all were significantly better at six, 12, and 24-months post first injection [32]. Significant improvements in Knee Injury and Osteoarthritis Outcome Score for Joint Replacement (KOOS-JR) scores were observed over time (F (4,12) =12.29, p<0.001) in a cohort. Following the procedure, clinical significance was accomplished at three, six, and 12-months following the procedure [16]. As evaluated by normalized KOOS, table 3 demonstrates the favorable changes in all five essential categories. All were much improved at six, 12, and 24 months after the first treatment [32]. Using all sample time points, the Sport Score and quality of life (QOL) score were nominally linked with an unadjusted p-value of 0.031 and 0.046, respectively [23].

**Table 3:** For 13 patients with KOA who were treated with BM-MSCs, a univariate analysis of normalized KOOS was performed KOA = knee osteoarthritis, BM-MSCs = bone marrow mesenchymal stem cells, KOOS = knee injury and osteoarthritis outcome score

Normalized KOOS sections	Baseline (mean)	6th-month follow-up (mean)	P-value	1-year follow-up (mean)	P-value	2 years follow-up (mean)	P-value
Symptoms	67.300	912.308	0.000	89.9	0.000	88.7	0.000
Pain	62.585	890.538	0.000	89.7	0.000	89.4	0.000
Daily life activity	64.223	908.308	0.000	92.2	0.000	93	0.000
Sport	40.25	799.769	0.000	81.1	0.000	81.6	0.000
Quality of life	34.162	754.923	0.000	76.9	0.000	77.4	0.000

Magnetic Resonance Imaging (MRI) Evaluation

A total of eight studies reported MRI evaluation at baseline and follow-up in the groups, including 659 patients [2,7,17,18,23,32,33,39]. Three studies [7,23,32] were followed for a total of 24 months, for 12 months, two studies [2,17] were followed up on, and two studies [33,39] were followed up for six months after they were completed, and one study [18], had a 48-weeks follow-up period. The transformation of the central medial femorotibial compartment (cMFTC) cartilage thickness [41] for a 24-month was −0.32 mm (SD=0.40) for those who have narrowed medial tibiofemoral joint and maintained knee pain at baseline in comparison to the control neither of which radiographic nor pain development (−0.12mm, SD=0.28) [7,42]. 67 percent of patients had progressed cartilage degeneration within the control group, with another 56 percent having extended osteophyte formation. Only 30% of individuals saw additional cartilage loss in the one-injection group, whereas 50% experienced osteophyte development advancement at 12 months. In the two-injection group, 89 percent of participants had cartilage improvement or no progression in cartilage loss, indicating that OA had stabilized, as seen by 89 percent of subjects having no progression in osteophyte formation [35]. The size of the cartilage defect in the MSCs group did not change substantially on MRI at six months (p =.5803), but the size of the cartilage defect in the control group grew significantly (p =.0049). Furthermore, the change in cartilage defect following the injection was significantly different between the two groups (p =.0051) [39]. Using the WORMS technique, the low-dose group had a mean change from baseline of -0.36 and -0.86 in both the left and right knees at week 48. Furthermore, the mean changes in total cartilage volume, knee femur end cartilage volume and knee patellar cartilage volume in the low-dose group were 54.58, 38.63, and 39.69 mm³, respectively. The knee tibial end cartilage volume and knee cartilage volume in the medium-dose group improved by 243.32 and 34.44 mm³, respectively. Increases of -0.42 and 122.92 mm³ in the left knee WORMS and knee femur end cartilage volume were reported in the high-dose group [18].

Two bilateral intra-articular knee injections, three weeks apart (18-20 days), were used in this preclinical study with AlloJoin. Because the high prevalence of bilateral KOA in the treatment population was investigated [18,43,44]. MRI showed no significant change in cartilage thickness after six months. As indicated in Table 4, there was a considerable improvement in knee cartilage thickness in the femoral and tibia plates after 12 months [32]. Time 2 (T2) scores in the patella region increased by a negligible amount (p =.055 for a two-sided test, nonadjusted). T2 changes (from baseline to 12 months) did not differ across the one, 10, or 50 million BM-MSCs cohorts [23]. The 50 million BM-MSCs doses (effect estimate [B] = 1.828, p =.002) maintained synovitis at lower levels than the one million BM-MSCs dose, according to statistical analysis of the effects of dose adjusted for both time and baseline levels of synovitis [23]. We found a decrease in pro-inflammatory monocytes/macrophages in synovial fluid three months after MSCs infusion, suggesting a potential mechanism of action. We do not see statistical significance relative to baseline levels (p =.062) because of the small number of patients who presented synovial fluid at baseline and three months after MSC infusion (n = 5). However, this downregulation suggests a potential mechanism of action of MSCs in the arthritic joint [23].

**Table 4:** Changes in knee cartilage thickness after the first injection after 12 months Mean baseline in mm = 2.15; mean at 12 months in mm = 2.45 T = time, SD = standard deviation, SE = standard error, P-value = probability value

Variable	Baseline (T1)	SD	SE	After 12 months of treatment (T2)	SD	SE	P-value (two-tailed)
Mean tibial plate thickness in mm	2.15	0.67	0.076	2.38	0.63	0.072	0.000
Mean femoral plate thickness in mm	2.16	0.78	0.09	2.5	0.76	0.086	0.000

Visual Analogue Scale (VAS)

A total of five studies [2,17,18,33,39] reported VAS evaluation at baseline and follow-up in the groups, including 194 patients. Two studies [2,17] were followed up for 12 months, two studies [33,39] were followed up for six months, and one study [18] was followed up for 48 weeks. VAS≤32 [7], (P < .00001) [10], (p ≤ 0.005) in Progenza (PRG) combined group [2]. In the MSCs group exclusively, the VAS for knee discomfort dropped dramatically from 6.8 0.6 to 3.4 1.5 (p.001) [39]. Our VAS data confirmed clinical improvement with these cell injections, as seen by the study's reported VAS minimal clinical improvement differences (MCID) score of 30.0 mm [18,45,46].

Western Ontario, and McMaster Universities Osteoarthritis Index (WOMAC) 

A total of six studies [2,17,18,23,35,39] reported WOMAC [47], evaluation at baseline, and follow-up in the groups, including 160 patients. Three studies [2,17,35] were tracked for 12 a year, one trial [23] was monitored for 24 months, one study [18] had a 48-weeks follow-up period, and for six months, one trial [39] was followed. (All P values were less than .05) [10]. Also, compared to the HA group, significantly more individuals had a 50% improvement in WOMAC, and after 12 months, the Re-Join® group had a 70% improvement rate, indicating that more patients were improving [17]. 

At six months after injection, a single injection of AD-MSCs resulted in a 55 percent reduction in the WOMAC total score, a 59 percent reduction in the WOMAC pain score, a 54 percent reduction in the WOMAC stiffness score, and a 54 percent reduction in the WOMAC physical function score [39]. According to a study in previous research [24,48-50], clinical outcomes improved six months following MSCs injection. The findings of this investigation support this. Furthermore, similar to earlier research [49,50], even six months following injection, the clinical outcomes were still good. This finding implies that with a single intra-articular MSCs injection, symptom alleviation can be sustained for up to six months [39]. Improvements in short form 36 (SF-36), -23.71 in WOMAC total, -17.14 in WOMAC-function, -2.29 in WOMAC stiffness, and -4.29 in WOMAC-pain were seen in the low-dose cohort. Improvements in left knee VAS were -2.25, right knee VAS was -2.13, WOMAC-total was -16.50, WOMAC-function was -11.88, WOMAC-stiffness was -1.71, and WOMAC-pain was -3.25 in the medium-dose cohort. The high-dose cohort observed statistically significant improvements in the left knee VAS of -1.36 and the right knee VAS of -2.07 [18]. The MCID averages for the WOMAC with KOA have been published [51]. The WOMAC functional score ranges between 9.1 to 19.9 mm, indicating that the WOMAC scores in this trial indicated considerable clinical improvement for the overall WOMAC functional (17.1) for both the left and right knees after 48 weeks for two of the doses [18,52-55].

Adverse Events (AEs)

A total of four studies [7,16,17,32] reported AEs evaluation at baseline and follow-up in the groups, including 550 patients. Two studies [7,32] were followed up for 24 months, and the others [16,17] were followed up for 12 months. Patient satisfaction was high (range: 8.1±2.1-8.8±1.9). All the patients said they would recommend the treatment to a friend, and 85 percent said they would do it again [16]. In the MSCs group, 10 (83%) patients experienced AEs, compared to seven (58%) individuals in the control group. No significant AEs or grade 4 or 5 AEs on the National Cancer Institute-Common Terminology Criteria for Adverse Events (NCI-CTCAE) scale. All the grade 3 AEs on the NCI-CTCAE scale were arthralgia, which completely disappeared within three days [39,56]. In the low-, middle-and high-dose groups, the incidence of AEs was 71.42 percent (5/7), 87.50 percent (7/8), and 100 percent (7/7), correspondingly [18].

Discussions

We evaluated the clinical efficacy and safety of intra-articular injection of MSCs in this study by thoroughly analyzing six RCTs and four clinical trials. The study's first strength is its comprehensiveness, a compilation of all current high-quality studies. Second, we assessed the included studies' cell adherence, cell immunophenotype, and cell differentiation ability using the MSC criteria established by the Mesenchymal Stem Cell Committee of the International Society for Cell Therapy (ISCT), and discovered that half of them meet the minimum requirements [16,18,23,35,39], as shown in table 1. Third, it contains tight inclusion and exclusion rules. Concurrent therapy studies, such as HA and PRP were omitted. The addition of newly incorporated research of AT and BM sources, we believe, is what has led to the divergent results. This is one of the reasons we are so adamant about completing this research. Compared to the control group, the MSCs group showed a considerable increase in cartilage volume.

The selection of the appropriate donor source and the optimal dose has become an essential issue due to the extensive research into MSCstherapy. BM, AT, placenta, and umbilical cord are among the most popular donor sources for MSCs in clinical research. Initially, people preferred to cultivate and expand BM-MSCs. Later research discovered that AT was more accessible than BM, had a simpler isolation technique, a larger yield, and the same chondrogenic capacity [10,57,58]. 

A reduction in pain is connected to the ability of cells to release bioactive chemicals. These elements are hypothesized to change the inflammatory milieu in the joint from pro-inflammatory to anti-inflammatory. PRG includes a high concentration of these bioactive substances in the cell culture supernatant, unlike other cell therapies. PRG may decrease the progression of OA based on the favorable cartilage outcomes from preclinical and clinical investigations. Many studies have found that beneficial effects are primarily apparent in the lateral tibial region. Although OA affects the entire joint, it has been hypothesized that the medial tibiofemoral region is more severely damaged than the lateral tibiofemoral region. As a result, because the medial tibiofemoral region is later, there may be fewer opportunities to demonstrate progress [2]. 

MPCs tagged with fluorescent dye lasted locally in the joint for up to 10 weeks in preclinical rat studies before becoming undetectable [18,59]. Furthermore, the serious adverse events (SAEs) contradict all preclinical animal investigations that revealed no evidence of systemic exposure [18,59-61]. In addition, earlier research has shown that Re-Join® is beneficial in rabbit and sheep models of OA [17,60,61]. The repair of osteoarthritis in rabbits and goats appears to be mediated by paracrine effects involving the stimulation of endogenous repair systems [26,32]. In a systematic evaluation of MSCs therapies, Lalu et al. found no significant side effects [23,62,63]. Following the aspiration of BM, there were no systemic side effects observed, and there were no issues that were noted [23]. Therefore, no individuals dropped out of the study [2].

Our findings show that there are statistically significant improvements in pain and function [2,7,10,16-18,33,35]. The average percentage of patients who have passed the Patient-Acceptable Symptom State (PASS) [64] the threshold was 35% in the placebo cohort(ranging from 33.1 to 35.5) and 48% in the intervention cohorts (varying between 42.2% to 56.1%) [7,65,66]. There were also decreases in present, typical, best, and worst numerical rating scale (NRS) pain [67], scores statistically significant over time (F(4,12)=14.5, p<0.001; F(4,12)=17.5, p<0.001; F(4,12)=2.9, p=0.003; and F(4,12)=35.5, p<0.001, respectively) [16]. Also, NRS pain in both the single and two injection protocol treatment groups, when compared to baseline, within-group improvement was statistically significant (0.05) at all time intervals [35]. Therefore, we found that all statistical tests for pain and functional outcome measures (n = 21) had a mean power of 0.877 15 SD [35]. The NPRS improved by 69 percent from baseline to the last follow-up at 12 months in both therapy groups. In comparison, arthroscopic debridement resulted in a 14 percent improvement in pain scores after 12 months, while a prescribed exercise regimen resulted in a 12 percent improvement in pain scores [35,68,69]. The range of motion in the MSCs group improved considerably from 127.9 10.3 to 134.6 12.5 at six months after injection (p =.0299) [39]. When these established MCID values were applied at 48 weeks, there was a reduction in pain and an improvement in knee function; however, due to the small number of participants included in this pilot investigation, these findings should be regarded with caution [18].

In addition, they discovered a link between the number of cells injected and pain relief [33]. Furthermore, two RCTs were recently reported, revealing significant improvements in pain and function in KOA patients after injection of autologous AD-MSCs versus controls [33]. MSCs generated from autologous BM showed a significant increase in clinical ratings [33,39]. Because the researchers differ in study design, cell type, supplementary therapy, and rehabilitation methods, it is difficult to determine the true differences in intra-articular injections of BM-MSCs and AD-MSCs [39].

Data reveal that one or more outcomes, such as KOOS pain, have improved statistically significantly [23,32,35], symptoms, SF-36 [18], VAS [2,10,16,18,33,39], and QOL scores [17,23,33], as well as WOMAC stiffness [2,10,16-18,23,33,35,39]. NPRS improved [16,35], from baseline to final follow-up at 12 months, by a percentage of 69 percent previous clinical trials have shown that intra-articular MSCs treatment can slow the course of OA [35]. All symptoms decreased dramatically, resulting in a considerable improvement in the quality of life of these grade 2 to 4 KOA patients. There is also evidence of safety. However, more research is required. Another concern is that most research focuses on short-term safety rather than long-term results [32]. Starting three months after the procedure, KOOS-JR scores improved dramatically, with clinically meaningful improvements lasting 12 months [16]. Within 48 weeks of follow-up, MCID scores for SF-36 are approximately 10%, which this study's data has surpassed [18,53,70,71]. Both groups improved significantly in Emory Quality of Life (EQOL), VAS, and all KOOS indicators pre-and post-procedure (p < .001) [33]. During follow-up, the two treatment groups' EQOL ratings altered in similar ways (similar temporal patterns across time) (p =0.98, test for interaction between time on study and treatment group) [33].

We report putative chondroprotective benefits and decreased synovial inflammation, with the 50 million cell dosage potentially being more beneficial. However, when compared to the 50 million and/or 10 million BM-MSC dosages, serum carboxy-terminus of the three-quarter peptide from cleavage of C I and C II (C1, C2), urine type II collagen cleavage neoepitope (C2C), and C-telopeptide of type II collagen (CTX-II) all increased significantly, suggesting a chondroprotective MSCs dose effect, as previously described [23]. Furthermore, exploratory MRI analyses of average cartilage volumes and average WORMS from baseline at week 48 revealed no change in the medium-dose (2*107 cells) and high-dose (5*107 cells) groups but an improvement in the low-dose AlloJoin (1*107 cells) group [18]. Over radiography x-rays, MRI assessments offered a more accurate picture of articular cartilage deterioration and change in location of the menisci [18,72]. Because MOAKS [73] is a semi-quantitative metric, the MRI analysis is limited [18]. Furthermore, MOAKs analysis demonstrating effective stabilization despite continuous bone marrow lesions (BMLs)contrasts with previous research that has found a link between BMLs and OA progression [35].

Because Orozco et al. showed a consistent improvement in cartilage quality during a two-year follow-up period from the baseline, we expect cartilage improvement in our series over a longer follow-up time [39,48]. Our research also saw increased cartilage volume and quality [2,17,18,23,32,39]. Furthermore, an MRI examination at 48 weeks revealed no signs of ectopic bone development [18]. Intra-articular injections of Re-Join® were found to enhance cartilage volume, with a significant rise 12 months after injection, suggesting that this could be a viable therapeutic intervention and cartilage regeneration for OA patients [17]. 

We believe that the subsequent trials should be greater [23]. The following trials should, in our opinion, be larger [18] and also look at the MSCs dose and the MSCs source. The safety of allogeneic MSCs for KOA must be established [23,32,39]. The usage of allogenic MSCs can be standardized, the dose can be more precisely regulated, and cell variability may be minimized. We should also examine the efficacy of BM and AD-derived orthobiologics treatments to develop a reliable judgment on which is the better choice for treating KOA [33]. MSCs, we feel, has the potential to be a definitive treatment for KOA [32]. It is also critical to distinguish the findings of this study from those of previous studies that used more various cell-based products, such as stromal vascular fraction [35].

This research has several limitations. The results should be treated with care first and foremost. We did our utmost to avoid simultaneous surgical treatment affecting efficacy. Second, all the studies we looked at used intra-articular injections. MSCs implantation by open or arthroscopic surgery has been proven to be more conducive to cartilage repair in several studies. While MSCs transplantation on a scaffold may help rebuild the anterior cruciate ligament and meniscus [10]. Third, four of our studies [16,23,32,33], were not RCTs. Fourth, we included three studies [23,33,39] that included KL grade 4 KOA patients. We do not know if the disease can be slowed or even reversed at this point in the disease's progression, especially using autologous-derived MSCs. Furthermore, as the human body ages, MSCs' ability to self-renew and differentiate decreases; particularly, the potential of MSCs in individuals with OA is lower than that of healthy persons [10,17,23,33,35]. 

## Conclusions

Our findings suggest that intra-articular injection of MSCs can reduce pain and enhance function in patients with KOA in a short period while also being relatively safe, even in the late stages of OA of the knee. Although there is inadequate evidence to suggest that MSCs may heal cartilage abnormalities at this time, we have reason to believe that they protect cartilage and slow down the deterioration of articular cartilage. These findings show that MSCs treatment has a bright future, but additional research and more homogeneous RCTs are needed to confirm it.
